# Genetic Characterization of Goutanap Virus, a Novel Virus Related to Negeviruses, Cileviruses and Higreviruses

**DOI:** 10.3390/v6114346

**Published:** 2014-11-12

**Authors:** René Kallies, Anne Kopp, Florian Zirkel, Alejandro Estrada, Thomas R. Gillespie, Christian Drosten, Sandra Junglen

**Affiliations:** 1 Institute of Virology, Medical Centre, University of Bonn, Sigmund-Freud-Str. 25, 53127 Bonn, Germany; E-Mails: rene.kallies@ufz.de (R.K.); kopp@virology-bonn.de (A.K.); zirkel@virology-bonn.de (F.Z.); drosten@virology-bonn.de (C.D.); 2 Estación de Biología Tropical Los Tuxtlas, Instituto de Biología, Universidad Nacional Autónoma de México, Apdo 176, San Andres Tuxtla, Veracruz, Mexico; E-Mail: aestrada@primatesmx.com; 3 Department of Environmental Sciences and Program in Population Biology, Ecology and Evolution, Emory University, Atlanta, GA 30322, USA; E-Mail: thomas.gillespie@emory.edu; 4 Department of Environmental Health, Rollins School of Public Health, Emory University, Atlanta, GA 30322, USA

**Keywords:** negevirus, insect-specific viruses, mosquito, next generation sequencing, virus taxonomy

## Abstract

Pools of mosquitoes collected in Côte d’Ivoire and Mexico were tested for cytopathic effects on the mosquito cell line C6/36. Seven pools induced strong cytopathic effects after one to five days post infection and were further investigated by deep sequencing. The genomes of six virus isolates from Côte d’Ivoire showed pairwise nucleotide identities of ~99% among each other and of 56%–60% to Dezidougou virus and Wallerfield virus, two insect-specific viruses belonging to the proposed new taxon Negevirus. The novel virus was tentatively named Goutanap virus. The isolate derived from the Mexican mosquitoes showed 95% pairwise identity to Piura virus and was suggested to be a strain of Piura virus, named C6.7-MX-2008. Phylogenetic inferences based on a concatenated alignment of the methyltransferase, helicase, and RNA-dependent RNA polymerase domains showed that the new taxon Negevirus formed two monophyletic clades, named Nelorpivirus and Sandewavirus after the viruses grouping in these clades. Branch lengths separating these clades were equivalent to those of the related genera *Cilevirus*, *Higrevirus* and *Blunervirus*, as well as to those within the family *Virgaviridae*. Genetic distances and phylogenetic analyses suggest that *Nelorpivirus* and *Sandewavirus* might form taxonomic groups on genus level that may define alone or together with *Cilevirus*, *Higrevirus* and *Blunervirus* a viral family.

## 1. Introduction

Mosquitoes transmit a great diversity of viruses that can cause severe disease in humans, e.g., Yellow fever virus, Dengue virus, and Chikungunya virus [1,2,3]. Pathogens that can replicate in mosquitoes and vertebrates, so called arboviruses, are members of six virus families, *Bunyaviridae*, *Flaviviridae*, *Togaviridae*, *Reoviridae*, *Rhabdoviridae* and *Asfarviridae*. Within recent years, novel viruses that seem to infect insects only and not to be able to replicate in vertebrates have been discovered in mosquitoes. These insect-specific or insect-restricted viruses belong not only to viral families that contain arboviruses [4,5,6,7,8,9,10,11,12] but also to clades in large phylogenetic distance to established families [13,14,15] suggesting that an even larger genetic diversity of insect-specific viruses exists in mosquitoes. Understanding the ecology and evolution of these viruses will also help to shed light on the evolution of arboviruses. Moreover, studies are needed that investigate the influence of insect-specific viruses on the replication and transmission of arboviruses.

One of these new taxa of insect-specific viruses is the recently proposed new taxon Negevirus [15]. Negeviruses were isolated from mosquitoes and phlebotomine sand flies collected in North and South America, Africa and Asia [15,16,17]. Viral particles are spherical with a size of 45 to 55 nm in diameter [15]. The single-stranded positive sense RNA genome is approximately 9 to 10 kb in length and comprises three open reading frames (ORF) that are flanked by untranslated regions and are separated by intergenic regions. The large ORF1 is predicted to represent the replicase gene. The encoded protein contains methyltransferase, ribosomal RNA methyltransferase, helicase and RNA-dependent RNA polymerase (RdRp) domains. ORF2 encodes a protein with membrane-spanning domains that may function as a structural protein. The role of the putative protein encoded by ORF3 is unknown. Phylogenetic analyses identified the plant-infecting Citrus leprosis C virus (CiLV-C) as closest relative. CiLV-C is transmitted by mites and is the only member of the genus *Cilevirus* (unassigned family) [18]. Other related plant infecting viruses are Hibiscus green spot virus (HGSV), the type species of the genus *Higrevirus* (unassigned family), and Blueberry necrotic ring blotch virus (BNRBV), proposed to be the type species of the genus *Blunervirus* (unassigned family) [19]. Phylogenetic analyses of the RdRp indicated similarities of CiLV-C, HGSV and BNRBV to the plant-infecting family *Virgaviridae* [19,20,21,22,23].

Here, we report the detection of a novel virus, named Goutanap virus (GANV), after the village Gouléako and Taï National Park in Côte d’Ivoire from which the mosquitoes originated, as well as of 33 strains of Piura virus (PIUV) from mosquitoes collected in Mexico.

## 2. Materials and Methods

### 2.1. Mosquito Collection and Virus Isolation

Mosquitoes were collected in the area of the Taï National Park in Côte d’Ivoire [24] and of the Palenque National Park in Mexico [25] and used for virus isolation as described [26]. Mosquitoes were identified using the identification keys of Darsie (2005) [27], Carpenter (1955) [28], and Clark-Gil (1983) [29] for the Mexican mosquitoes and the identification keys of Jupp (1996) [30], Edwards (1941) [31], Gillies de Meillon (1968) [32] and Gillies and Coetzee (1987) [33] for the African mosquitoes, respectively. In total, 371 pools consisting of 3491 mosquitoes collected in Mexico and 432 pools containing 4839 mosquitoes collected in Côte d’Ivoire were analysed. Several other viruses have been identified in the same material [7,8,9,11,24,25,26,34,35]. Briefly, mosquitoes were pooled according to species or genus [25,26] and homogenized. *Aedes albopictus* (C6/36) and African green monkey (Vero E6/7) cells were infected with mosquito homogenates and observed for cytopathic effects (CPE). CPE positive cultures were used to infect confluent C6/36 cells in 175 cm^2^ cell culture flasks. Virus particles were pelleted by ultracentrifugation through a 36% sucrose cushion, re‑suspended in 200 µL PBS and stored at −80 °C until further analysis.

### 2.2. Nucleic Acid Extraction and Next Generation Sequencing

Viral nucleic acids from Ivorian samples were extracted using TRIzol (Life Technologies, Darmstadt, Germany) and double-strand cDNA was synthesized using the cDNA Synthesis System Kit (Roche, Mannheim, Germany) following the manufacturer’s instructions. One hundred nanograms (ng) of cDNA per sample were fragmented as described in the Ion Xpress Plus gDNA Fragment Library Preparation Manual (Life Technologies). Fragment ends were repaired, barcode containing adapter oligonucleotides were ligated and emulsion PCR (emPCR) was performed according to the Ion Torrent protocol (Life Technologies). Next Generation Sequencing (NGS) was performed on a 316 chip using the Ion Torrent platform (Life Technologies). Viral nucleic acids from Mexican samples were extracted using the RNeasy Mini Kit (Qiagen, Hilden, Germany) and cDNA synthesis was performed with the Superscript OneCycle cDNA Kit (Life Technologies) according to the manufacturer’s instructions. Fragmentation of 100 ng cDNA per sample was done by nebulization and a library was constructed according to the GS Junior Titanium Series Rapid Library Preparation Method Manual (Roche). Fragment end repair, adaptor ligation and emPCR (Kit Lib-L) were done following the standard Roche protocols. NGS was performed on a Roche 454 GS Junior system.

### 2.3. Analysis of NGS Reads and Phylogenetic Analyses

NGS reads were assembled in Geneious v6 and contigs were aligned against the GenBank virus database using the blastn and blastx algorithms [36]. Conserved protein domains were identified using the Conserved Domain Database webserver [37]. For calculation of nucleotide (nt) identity matrices nucleotide sequences were aligned using ClustalW [38]. For phylogenetic analyses concatenated amino acid (aa) alignments of the methyltransferase, the helicase and the RdRp conserved protein domains of all available negeviruses, CiLV-C, HGSV, BNRBV and of representative members from all genera of the family *Virgaviridae* (Table S1) were aligned using MAFFT v7 [39]. Phylogenetic trees were inferred in PhyML on a gap-free alignment with the Blosum62 and Dayhoff substitution matrix and 1000 bootstrap replications [40].

### 2.4. PCR Screening

RNA was extracted from cytopathic cell culture supernatants that were inoculated with homogenates of the Mexican mosquitoes using the Nucleo spin RNA virus Kit (Machery and Nagel, Düren, Germany). cDNA was synthesized using random hexamer primers and Superscript III Reverse transcriptase (Life Technologies). PCR was performed using Platinum Taq polymerase (Life Technologies, Darmstadt, Germany), the primers MX_C6.7_F1 (5'-CTTGAGATTAAGTTCTTCGAGTTTGAGC) and MX_C6.7_R1 (5'-AAAGGCGGTGTGATCGGTG) and 1 µL template cDNA. PCR products were visualized by agarose gel electrophoresis and Sanger sequenced (SeqLab, Göttingen, Germany).

### 2.5. Nucleotice Sequence Accession Numbers

The genome sequences of Goutanap virus were assigned to GenBank accession numbers KM249339 and KF588035 to KF588039. The genome sequence of Piura virus C6.7-MX-2008 was assigned to GenBank accession number KM249340. Sequence fragments of the RdRp domain of other Piura virus isolates were assigned to GenBank accession numbers KM258581 to KM258599 and KM924386 to KM924398.

## 3. Results and Discussion

Seven mosquito pools induced strong CPE one to five days post infection in C6/36 cells and were further investigated by NGS (Table 1). Genomes were assembled and compared to viral sequences in the NCBI database revealing that seven negevirus-like viruses were identified. Maximum pairwise nucleotide identities between 56% to 60% of the six isolates originating from Côte d’Ivoire were found to Wallerfield virus (WALV) and to Dezidougou virus (DEZV). Pairwise identities among the six novel genomes were 99.3% to 100% on nt level and 99.6% to 100% on aa level suggesting the detection of six isolates of one novel virus species. We designated this virus GANV. Genome organization of GANV was similar to WALV and DEZV and other negevirus-like viruses [15,16,17]. GANV genomes ranged between 9141 and 9170 nt in length and comprised three ORFs that were separated by intergenic regions and flanked by UTRs (Table 2). A poly-A tail at the 3'-end of the genomes was detected indicating a positive sense RNA genome. ORFs were further analyzed for conserved protein domains. Five domains were predicted for ORF1: a methyltransferase domain (nt 298 to 1377), a ribosomal RNA methyltransferase domain (nt 2392 to 2949), a helicase domain (nt 3985 to 4797), and a RdRp domain (nt 5431 to 6738) (nt positions refer to GANV F33-CI-2004). Similar to other negevirus-like sequences, no conserved domains were identified for ORF2 and ORF3.

**Table 1:** GANV and PIUV positive mosquito pools. Mosquitoes have been collected in Côte d’Ivoire (CI) in 2004 and in Mexico (MX) in 2008.

Virus	Isolate	Host Taxon	No. of Individuals	Trapping Locality
GANV	C68-CI-2004	*Culicidae* spp.	10	Secondary rainforest, CI
GANV	F33-CI-2004	*Culex nebulosus*	12	Gouléako, CI
GANV	F35-CI-2004	*Culex nebulosus*	16	Gouléako, CI
GANV	F47-CI-2004	*Culicinae* spp.	10	Taï, CI
GANV	F54-CI-2004	*Culex antenatus*	20	Taï, CI
GANV	F55-CI-2004	*Culex antenatus*	9	Taï, CI
PIUV	C6.7-MX-2008	*Mansonia* spp., *Wyeomyia* spp.	10	Palenque, MX
PIUV	A2.1-MX-2008	*Trichoprosopon* spp., *Coquillettidia* spp.	10	Palenque, MX
PIUV	A8.5-MX-2008	*Culex* spp.	10	Palenque, MX
PIUV	A11.18-MX-2008	*Culex* spp.	10	Palenque, MX
PIUV	A11.22-MX-2008	*Culex* spp.	10	Palenque, MX
PIUV	B1.2-MX-2008	*Psorophora* spp.	10	Palenque, MX
PIUV	B1.3-MX-2008	*Culex* spp.	10	Palenque, MX
PIUV	B1.4-MX-2008	*Culex* spp., *Trichoprosopon* spp.	10	Palenque, MX
PIUV	B1.5-MX-2008	*Culex* spp., *Trichoprosopon* spp.	10	Palenque, MX
PIUV	B1.6-MX-2008	*Culex* spp., *Wyeomyia* spp.	10	Palenque, MX
PIUV	B2.3-MX-2008	*Culex* spp., *Wyeomyia* spp., *Psorophora* spp.	10	Palenque, MX
PIUV	B5.21-MX-2008	*Wyeomyia* spp., *Trichoprosopon* spp.	10	Palenque, MX
PIUV	B8.9-MX-2008	*Culex* spp.	10	Palenque, MX
PIUV	B8.10-MX-2008	*Culex* spp.	10	Palenque, MX
PIUV	B8.11-MX-2008	*Culex* spp.	10	Palenque, MX
PIUV	B8.12-MX-2008	*Culex* spp.	10	Palenque, MX
PIUV	B8.13-MX-2008	*Culex* spp.	10	Palenque, MX
PIUV	B8.14-MX-2008	*Culex* spp.	10	Palenque, MX
PIUV	B10.1-MX-2008	*Culex* spp., *Psorophora* spp., *Mansonia* spp., *Toxorhynchites* spp.	9	Palenque, MX
PIUV	B10.2-MX-2008	*Culex* spp.	10	Palenque, MX
PIUV	B10.3-MX-2008	*Culex* spp.	10	Palenque, MX
PIUV	C1.20-MX-2008	*Culex* spp.	10	Palenque, MX
PIUV	C1.22-MX-2008	*Culex* spp.	10	Palenque, MX
PIUV	C1.24-MX-2008	*Culex* spp.	10	Palenque, MX
PIUV	C1.25-MX-2008	*Culex* spp.	2	Palenque, MX
PIUV	C5.2-MX-2008	*Mansonia* spp., *Wyeomyia* spp.	10	Palenque, MX
PIUV	C6.6-MX-2008	*Culex* spp.	9	Palenque, MX
PIUV	C6.9-MX-2008	*Mansonia* spp.	10	Palenque, MX
PIUV	C7.1-MX-2008	*Culex* spp., *Aedes* spp. *, *Toxorhynchites* spp.	10	Palenque, MX
PIUV	C7.2-MX-2008	*Culex* spp., *Mansonia* spp., *Wyeomyia* spp.	10	Palenque, MX
PIUV	D2.1-MX-2008	*Culex* spp.	10	Palenque, MX
PIUV	D2.2-MX-2008	*Culex* spp.	10	Palenque, MX
PIUV	D2.3-MX-2008	*Culex* spp.	8	Palenque, MX

* Based on the old classification system.

Isolate C6.7-MX-2008 originating from Palenque, Mexico showed 94.8% nt identity and 98.4% aa identity to PIUV P60 from Peru [15] and was thus suggested to be a novel strain of PIUV, named PIUV Palenque C6.7-MX-2008. Genome organization of PIUV Palenque C6.7-MX-2008 is shown in Table 2. RT-PCR screening of additional cytopathic cell cultures inoculated with Mexican mosquitoes yielded 32 cultures also infected with PIUV (Table 1). Sequence fragments showed 95.3% to 100% nt identity and 97.2% to 100% aa identity among each other indicating that all isolates were strains of PIUV. Infected mosquito pools mainly contained *Culex* mosquitoes but also species of the genera *Aedes*, *Mansonia, Psorophora*, and *Wyeomyia* showing that PIUV infects a large diversity of mosquito species (Table 1). Thus far, negevirus-like viruses were only identified in mosquitoes of the genera *Anopheles*, *Culex*, *and Armigeres* [15,16,17]. The infection rate was with 32% (33/102 pools) extremely high. No other studies investigating the negevirus infection rate in mosquitoes have been reported so far. However, the detection of these viruses in several countries on different continents suggests a wide distribution and a high prevalence in mosquito populations. It has recently been shown that the co‑infection of mosquitoes with an insect-specific flavivirus may enhance or suppress the transmission of a mosquito-borne flavivirus [41,42,43]. Thus, it will be of great interest to study if these viruses may influence infection, replication and transmission of arboviruses by mosquitoes.

**Table 2:** Genome organization of GANV and PIUV C6.7-Mx-2008.

Virus	Strain	Genome	5'UTR *	ORF1	Intergenic Region	ORF2	Intergenic Region	ORF3	3'UTR *
GANV	C68-CI-2004	9151	63	6732	31	1233	98	660	334
GANV	F33-CI-2004	9165	66	6732	31	1233	98	660	335
GANV	F35-CI-2004	9170	66	6732	31	1233	98	660	341
GANV	F47-CI-2004	9141	41	6732	31	1233	98	660	337
GANV	F54-CI-2004	9164	65	6732	31	1233	98	660	336
GANV	F55-CI-2004	9168	67	6732	31	1233	98	660	336
PIUV	C6.7-MX-2008	10018	716	7011	42	1203	140	618	288

* incomplete.

**Figure 1:**
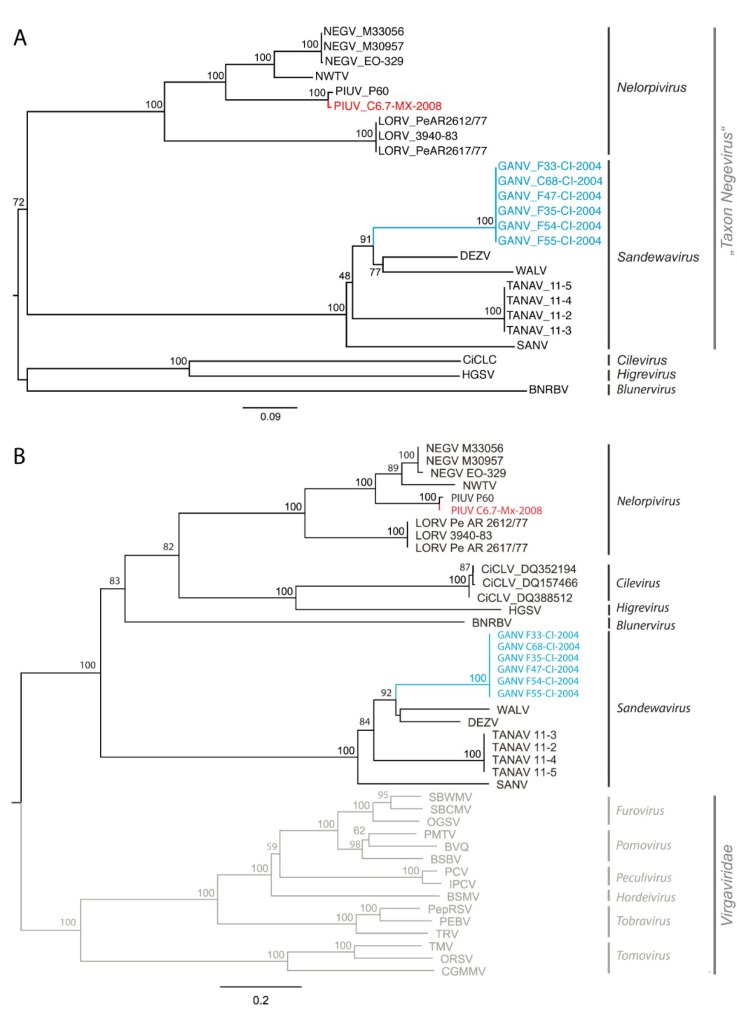
Phylogenetic relationship of Goutanap virus and Piura virus C6.7-MX-2008. (**A**) Analysis of the conserved domains of the RNA-dependent RNA polymerase proteins of negeviruses, cileviruses, higreviruses and blunerviruses; (**B**) Analysis of a gap-free concatenated alignment of fused methyltransferase, helicase and RNA-dependent RNA polymerase domains of members of negeviruses, cileviruses, higreviruses and blunerviruses, as well as representative members of each genus of the *Virgaviridae* family. ML analyses were based on an 805 amino acid alignment (**A**) and a 445 amino acid alignment (**B**) guided by the Blosum62 (**A**) and Dayhoff (**B**) substitution matrix using PHYML as implemented in Geneious. Confidence testing was performed with 1000 bootstrap replicates. Virus names and GenBank accession numbers are provided in Table S1.

Phylogenetic analyses based on conserved RdRp domains grouped GANV in a basal phylogenetic position to the two sister species WALV and DEZV (Figure 1A). The three viruses formed a monophyletic clade with Santana virus (SANV) and Tanay virus (TANAV), tentatively designated Sandewavirus after the first viruses that were described in this clade (from Santana, Dezidougou and Wallerfield). PIUV Palenque C6.7-MX-2008 clustered together with PIUV P60, Negev virus (NEGV), Ngewotan virus (NWTV), and Loreto virus (LORV) (Figure 1A). This clade was tentatively named Nelorpivirus after Negevirus, Loreto and Piura. Plant infecting viruses of the genera *Cilevirus*, *Higrevirus* and *Blunervirus* paired in sister relationship to all members of the taxon Negevirus with Blunervirus branching close to the tree root on a solitary branch. This topology was not contradicting the topologies presented by Auguste *et al.* (2014) [16], Nabeshima *et al.* (2014) [17] and Vasilakis *et al.* (2013) [15]. Intra- and intergenetic distances for the clades are shown in Table 3.

In an attempt to root the phylogeny we repeated the phylogenetic analysis based on concatenated protein sequences of the methyltransferase, helicase and RdRp domains including representative members of each genus of the *Virgaviridae*, a closely related family (Figure 1B). In this phylogeny using virgaviruses as an outgroup, the ingroup root was placed at a different location separating *Sandewavirus* from other negeviruses, as well as the genera *Cilevirus*, *Higrevirus* and *Blunervirus*.

**Table 3:** Intra- and interclade amino acid pairwise identity values of *Nelorpivirus*, *Sandewavirus*, *Cilevirus*, *Higrevirus* and *Blunervirus*. Classification into five clades was based on ML analyses as shown in Figure 1.

Clade	Nelorpivirus	Sandewavirus	Cilevirus	Higrevirus	Blunervirus
Nelorpivirus	54.1–72.1				
Sandewavirus	30.1–33.4	58.0–70.8			
Cilevirus	32.2–32.7	25.5–26.9	/		
Higrevirus	31.0–31.8	25.3–27.3	41.7	/	
Blunervirus	28.4–29.5	23.7–25.8	25.4	25.6	/

However, based on genome organization and host association, the topology in Figure 1A seems more likely than the topology in Figure 1B, as viruses with similar genome organizations and hosts cluster with each other in Figure 1A. The insect-infecting viruses have a genome consisting of one segment of positive-sense RNA while the genomes of plant-infecting cile-, higre-, and blunerviruses are two-, three-, and four-segmented, respectively (and double-stranded in case of *Blunervirus*). However, the clustering of *Blunervirus* with other plant-infecting viruses might as well result from a long-branch attraction effect that could resolve upon inclusion of further (as yet unknown) taxa on this solitary branch.

According to phylogenetic distances separating established genera within the family *Virgaviridae* the tree topology suggests that *Nelorpivirus* and *Sandewavirus* might form taxonomic groups on genus level. Differences in genome architecture may not suffice to define whether *Nelorpivirus* and *Sandewavirus* alone or in combination with *Cilevirus*, *Higrevirus* and *Blunervirus* define a viral family, as differences in the number of genomic RNAs of different genera are known in other viral families, e.g., *Viragviridae* [21,23] and *Reoviridae* [44].

In summary, phylogenetic analyses suggest that the taxon Negevirus as presented in Vasilakis *et al.* (2013) [15] is a monophyletic taxon in which several distinct genera and potentially higher taxonomic units exist. Our finding of GANV corroborates the existence of clearly separated subgroups within two major Negevirus clades (*Nelorpivirus* and *Sandewavirus*). All negeviruses share one type of genome organization (non-segmented, positive-sense ssRNA) as opposed to outgroup taxa, which in turn have heterogeneous genome organizations, predicting the taxon Negevirus to constitute a candidate virus family or subfamily. The high infection rate of *Nelorpivirus* and *Sandewavirus* in mosquito populations and their wide geographic distribution invites further studies on the influence of negeviruses on mosquito populations as well as the transmission of mosquito-borne disease.

## Supplementary Files

Supplementary File 1
