# Supplementary material for: Genetic Characterization of Goutanap Virus, a Novel Virus Related to Negeviruses, Cileviruses and Higreviruses
Source: Viruses. 2014 Nov 12;6(11):4346–57. doi: 10.3390/v6114346 (PMC4246226; doi:10.3390/v6114346)
Supplement: Supplementary File 1 [file viruses-06-04346-s001.pdf]

Supplementary Material

Genetic Characterization of Goutanap Virus, a Novel Virus Related to Negeviruses, Cileviruses and Higreviruses

René Kallies, Anne Kopp, Florian Zirkel, Alejandro Estrada, Thomas R. Gillespie, Christian Drosten and Sandra Junglen

Table S1. Viruses used in the phylogenetic analyses.

Family/Genus	Virus Species	Abbreviation	Strain	Accession Number
Unclassified/ unclassified	Dezidougou virus	DEZV	ArA 20086	JQ675604
	Loreto virus	LORV	3940-83	JQ675610
			Pe AR 2612/77	JQ675611
			Pe AR 2617/77	JQ675612
	Negev virus	NEGV	EO-329	JQ675605
			M30957	JQ675608
			M33056	JQ675609
	Ngewotan virus	NWTV		JQ686833
	Piura virus	PIUV		JQ675607
	Santana virus	SANV		JQ675606
	Tanay virus	TANAV	11-2	NC_024071
			11-3	KF425262
			11-4	KF425263
			11-5	KF425264
	Wallerfield virus	WALV		NC_023440
	Blueberry necrotic ring blotch virus	BNRBV		NC_016084<br>NC_016085
Unclassified/ *Cilevirus*	Citrus leprosis virus C	CiCLV		DQ352194
		CiCLV		DQ157466
		CiCLV		DQ388512
Unclassified/ *Higrevirus*	Hibiscus green spot virus	HGSV		NC_016141
Virgaviridae/ *Furovirus*	Oat golden stripe virus	OGSV		NC_002358
	Soil-borne cereal mosaic virus	SBCMV		NC_002351
	Soil-borne wheat mosaic virus	SBWMV		NC_002351
Hordaivirus	Barley stripe mosaic virus	BSMV		NC_003469
Peculivirus	Peanut clump virus	PCV		NC_003672
	Indian peanut clump virus	IPCV		NC_004729
Pomovirus	Beet soil-borne virus	BSBV		NC_003520
	Beet virus Q	BVQ		NC_003510
	Potato mop-top virus	PMTV		NC_003723
Tobamovirus	Cucumber green mottle mosaic virus	CGMMV		NC_001801
	Odontoglossum ringspot virus	ORSV		NC_001728
	Tobacco mosaic virus	TMV		NC_001367
Tobravirus	Pepper ringspot virus	PepRSV		NC_003669
	Pea early browning virus	PEBV		NC_002036
	Tobacco rattle virus	TRV		NC_003805
